# The Moral Dilemma of Euthanasia Through the Eyes of the Medical Society in Bulgaria

**DOI:** 10.7759/cureus.49615

**Published:** 2023-11-28

**Authors:** Ivan I Tsranchev, Biliana Mileva, Metodi Goshev, Pavel Timonov, Svetlozar Spasov, Alexandar Alexandrov

**Affiliations:** 1 Department of Forensic Medicine and Deontology, Medical University of Plovdiv, Plovdiv, BGR; 2 Department of Forensic Medicine and Deontology, Medical University of Sofia, Sofia, BGR; 3 Department of of Forensic Medicine, University Hospital St. George Plovdiv, Plovdiv, BGR

**Keywords:** euthanasia, perspectives on euthanasia, mercy killing, moral dilemma, physician-assisted suicide, bulgarian medical society, active euthanasia

## Abstract

Introduction: With the development of human society, the question of the value and inviolability of human life begins to occupy a central place in the various social strata and social structures. With the adoption of the Universal Declaration of Human Rights after the Second World War, the basic postulates protecting the right to inviolability of human life were laid. The question focused on euthanasia has been discussed in several European countries, such as Germany, Ireland, France, and Italy, leading to considerable interest in the medical community in Bulgaria.

Materials and methods: A prospective study was performed using approved sample cards, analyzing the general knowledge of the medical community in the Republic of Bulgaria about euthanasia and assisted suicide over a period of four months, between January 2023 and May 2023. In this process, 623 people were surveyed, and the questionnaire included several targeted questions through electronically generated samples on the Microsoft Forms platform. The target group had doctors with and without a specialty in various fields of hospital and pre-hospital care, dentists, and students from the fields of medicine and dentistry.

Results and discussion: The results show that the majority of medical professionals clearly state their positive opinion on the adoption of a law to legalize euthanasia in the Republic of Bulgaria, clearly taking into account the fact that the right to life has always been and always will be the most absolute and fundamental human right. Contrary to the above, it is implied that it is inevitably linked to a quality and fulfilling life without suffering. Identically, they also answered that a person should have the right to know exactly when to end his own life. The medical society in Bulgaria clearly shows its positive opinion regarding the idea that the different forms of euthanasia (active euthanasia and assisted suicide) should be defined as morally and legally permissible. Our research confirms the Bulgarian medical community's opinion that the subject of the problems of euthanasia and its legal regulation are already ripe for public discussion, similar to many other European countries.

Conclusion: The actual issue of euthanasia as a conclusion raises several questions related to the process of acceptance of standard algorithms for action in such cases where the same action is legalized by law. It also includes the process of acceptance of strict regulations by the countries for the so-called negative phenomenon "death tourism" and several other administrative actions related to the mandatory registration of every case of euthanasia, the implementation of mandatory consultations with a psychiatrist and psychologist for patients seeking euthanasia as the only possible option, and providing possible alternatives regarding their illness. This is the unchangeable cornerstone for standardizing the legalization process and acceptance of "good death" in Bulgaria. In its essence, euthanasia creates both a social and an ethical conflict in our modern society, appearing at the same time as a kind of "stress test" for the health system.

## Introduction

The supreme human right to life is absolute, and its nature guarantees it in several globally and publicly recognized legal documents. Philosophic thoughts that death is a direct continuation of the natural life cycle of every living being, including man as the highest representative in evolutionary development, have been discussed since ancient times, especially in the Egyptian, Sumerian, Babylonian, and other world cultures. With the development of human society, the question of the value and inviolability of human life begins to occupy its central place in the various social strata and social structures. With the adoption of the Universal Declaration of Human Rights after the Second World War, the basic postulates protecting the right to the inviolability of human life were laid [1]. This immutable human right, inherent in each of us since birth, is fundamental to every state constitution. Philosophically, on the same side of the coin is the right to a dignified life and quality of life for each of us. All this is in the bipolar antagonism of so many still incurable diseases that raises the fundamental question of euthanasia nowadays. This question has been discussed in several European countries, such as Germany, Ireland, France, and Italy [2-7], leading to a considerable interest in the medical community in Bulgaria.

## Materials and methods

For this scientific work, a prospective study was performed using approved sample cards, analyzing the general knowledge of the medical community in the Republic of Bulgaria about euthanasia and assisted suicide over a period of four months, between January 2023 and May 2023. In this process, 623 people were surveyed, and the questionnaire included several targeted questions through electronically generated samples on the Microsoft Forms (Microsoft Corp., Redmond, WA) platform. The target group had doctors with and without a specialty in various fields of hospital and pre-hospital care, dentists, and students from the fields of medicine and dentistry. The questions asked in the survey are summarized in Table 1.

**Table 1:** A sample of the questionnaire with 11 questions related to euthanasia and assisted suicide

What effect would the legalization of euthanasia have on the doctor-patient relationship?
Do you think that the legalization of euthanasia would lead to a cascade of negative consequences associated with this act by significantly increasing the wish among patients to end their lives?
Do you think that quality palliative care for patients can be an alternative to euthanasia?
Do you think that every single patient can make a correct decision about the exact point at which they want to end their life by the method of euthanasia?
From a religious point of view, would you approve of any act of euthanasia?
In your opinion, has the development of medicine reached the level of fully diagnosing and treating terminal conditions and deriving an objective assessment of the duration and quality of life of terminally ill patients?
Do you think there is a difference between euthanasia and assisted suicide?
In your opinion, will the adoption of a law legalizing euthanasia help the vulnerable groups of society and those groups of people with various incurable disabilities and diseases?
Do you think the right to life is the highest, inviolable, and absolute human right?
Do you think that a person should have the choice of when and in what way to end his life?
What do you think is morally and legally permissible as a possible action?

The statistical program SPSS version 12 (SPSS Inc., Chicago, IL) processed the data and presented it as pie diagrams.

## Results

**Figure 1:**
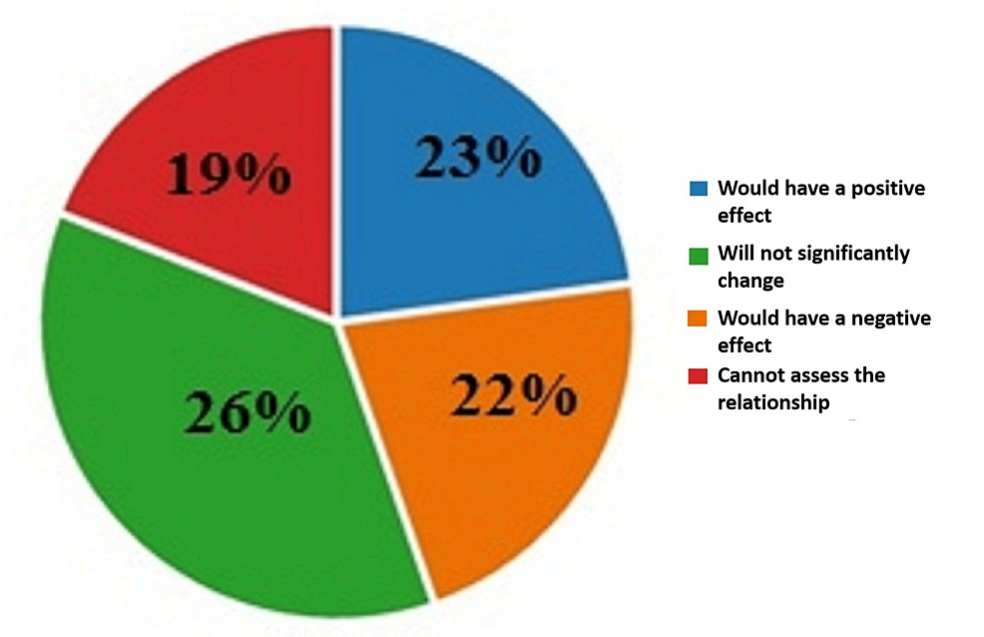
Answers by percentages to the first question: What effect would the legalization of euthanasia have on the doctor-patient relationship?

To the first question on the effect the legalization of euthanasia will have on the doctor-patient relationship, 23% (142) of the respondents answered that the legalization of euthanasia in Bulgaria would have a positive effect on the doctor-patient relationship; 22% (135) of the respondents had the opposite opinion on the matter; 26% (225) of them believed that the legalization of euthanasia in Bulgaria will not significantly change the relationship between doctor and patient; and 19% (118) answered that they cannot assess how this would affect this relationship. The results are illustrated in Figure 1.

**Figure 2:**
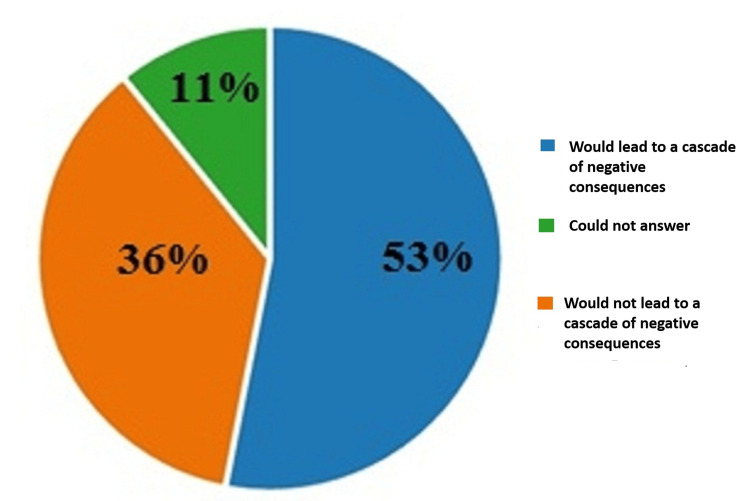
Answers by percentages to the second question: Do you think that the legalization of euthanasia would lead to a cascade of negative consequences associated with this act by significantly increasing the wish among patients to end their lives?

On the second question of the questionnaire on whether the legalization of euthanasia would lead to a cascade of negative consequences associated with this act by significantly increasing the wish among patients to end their lives, 53% (328) answered that they thought that the legalization of euthanasia would lead to a cascade of negative consequences related to an increase in the number of cases wishing to end their lives using euthanasia as a method; 36% (223) of the respondents had the opposite opinion, and 11% (67) of the respondents could not answer this question clearly. The results are illustrated in Figure 2.

**Figure 3:**
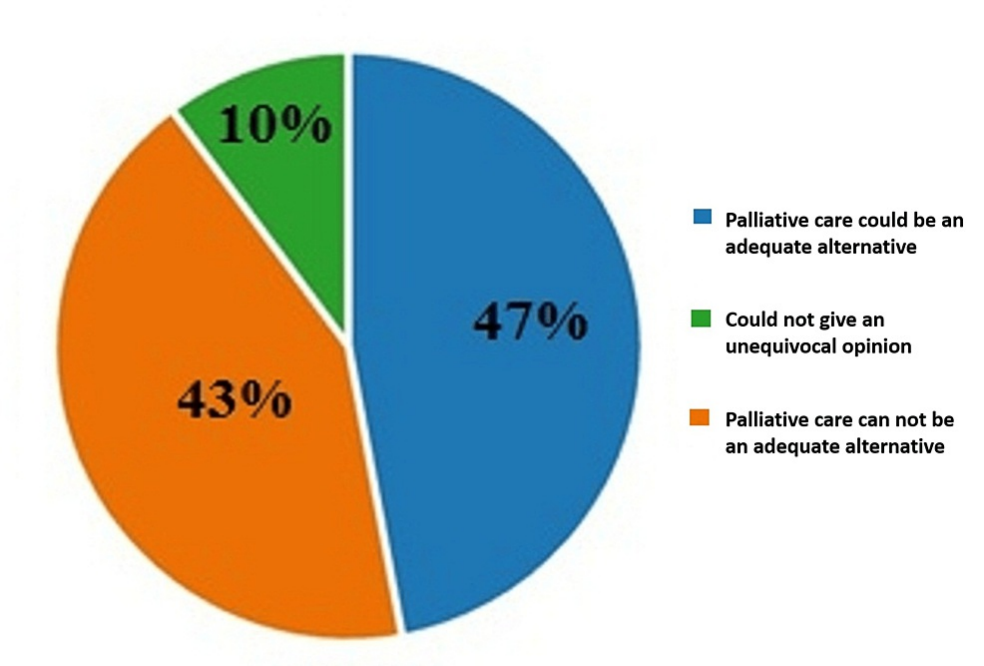
Answers by percentages to the third question: Do you think that quality palliative care for patients can be an alternative to euthanasia?

On the third question of the questionnaire which sought the participants' opinion on whether quality palliative care for patients can be an alternative to euthanasia, 47% (290) of the respondents believed that quality palliative care could be an adequate alternative to euthanasia; 43% (263) of the respondents were of the opposite opinion; and 10% (63) could not give an unequivocal opinion of the same question. The results are illustrated in Figure 3.

**Figure 4:**
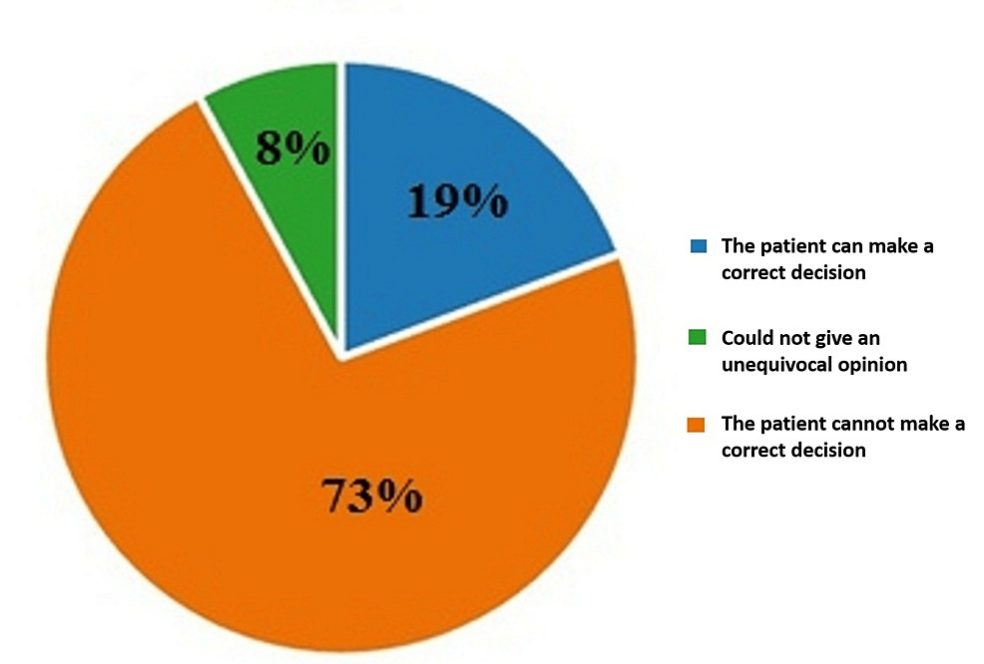
Answers by percentages to the fourth question: Do you think that every single patient can make a correct decision about the exact point at which they want to end their life by the method of euthanasia?

On the fourth question of the questionnaire, "Do you think every single patient can make a correct decision about the exact point at which they want to end their life by the method of euthanasia?", 73% (448) of the respondents believed that the patient could not make a correct decision about when to end his life by applying the method of euthanasia; 19% (119) of the respondents had the opposite opinion on the subject; and 8% (49) did not have an unequivocal opinion on the matter. The results are illustrated in Figure 4.

**Figure 5:**
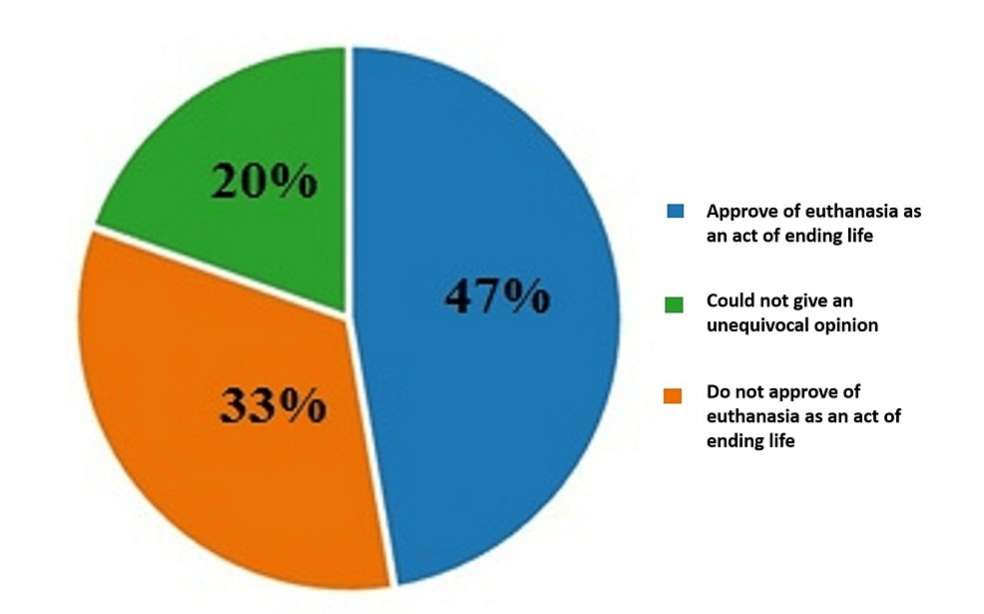
Answers by percentages to the fifth question: From a religious point of view, would you approve of any act of euthanasia?

On the fifth question which was about their approval of euthanasia from a religious point of view, 47% (293) of the respondents answered that, from a religious point of view, they approved of euthanasia as an act of ending life, 33% (205) expressed the opposite opinion, and 20% (121) of the respondents could not give an unequivocal opinion on the question. The results are illustrated in Figure 5.

**Figure 6:**
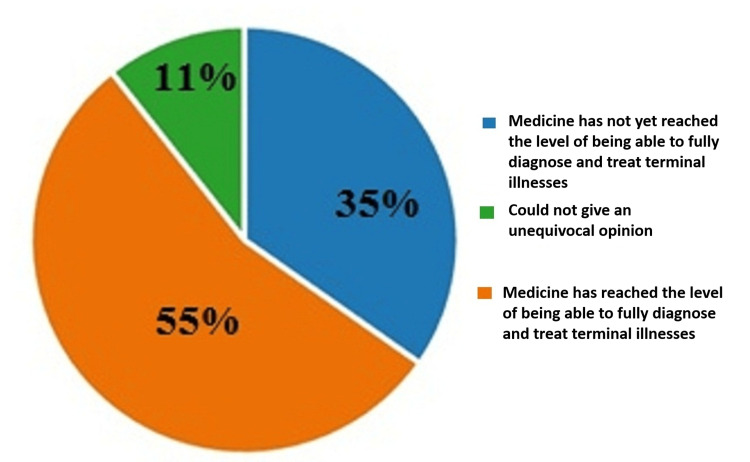
Answers by percentages to the sixth question: In your opinion, has the development of medicine reached the level of fully diagnosing and treating terminal conditions and deriving an objective assessment of the duration and quality of life of terminally ill patients?

On the sixth question "In your opinion, has the development of medicine reached the level of fully diagnosing and treating terminal conditions and deriving an objective assessment of the duration and quality of life of terminally ill patients?", 55% (215) thought that the development of medicine has yet to reach the level of being able to fully diagnose and treat terminal illnesses and derive an objective assessment of the duration and quality of life of terminally ill patients. On this topic, 35% (338) answered negatively, and 11% (66) could not give an unequivocal answer. The results are illustrated in Figure 6.

**Figure 7:**
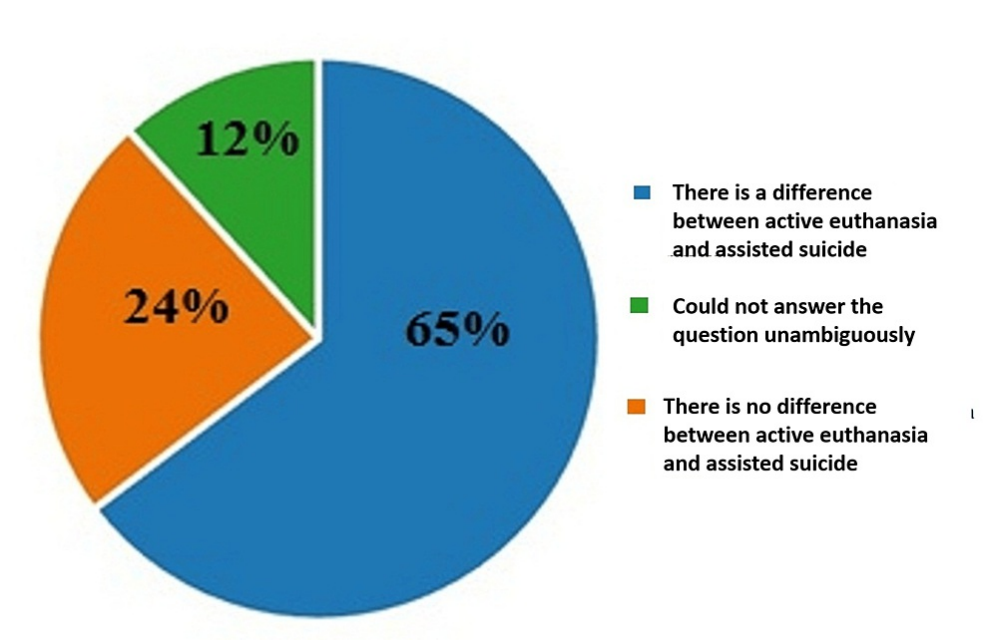
Answers by percentages to the seventh question: Do you think there is a difference between euthanasia and assisted suicide?

On the seventh question which was about their thoughts on the difference between euthanasia and assisted suicide, 65% (401) considered that there is a difference between active euthanasia and assisted suicide, 24% (146) answered negatively, and 12% (73) of the participants could not answer the question unambiguously. The results are illustrated in Figure 7.

**Figure 8:**
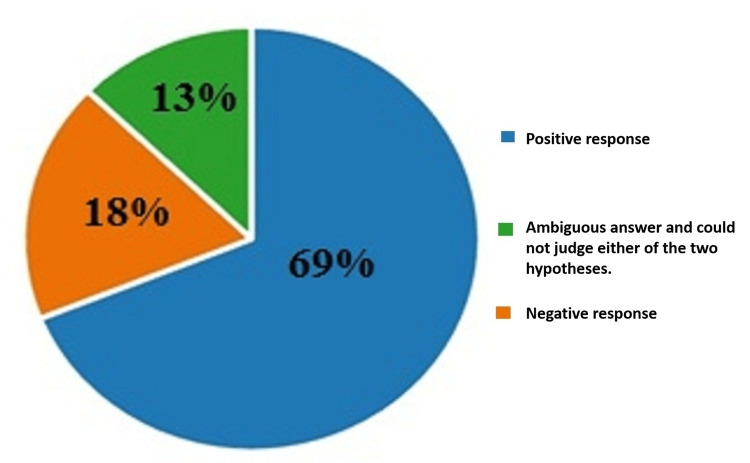
Answers by percentages to the eighth question: In your opinion, will the adoption of a law legalizing euthanasia help the vulnerable groups of society and those groups of people with various incurable disabilities and diseases?

On the eighth question concerning the opinion of the medical guild on the adoption of a law legalizing euthanasia and how it would help the vulnerable groups of society and those groups of people with various terminal disabilities and diseases, 69% (427) answered positively, 18% (114) of the respondents answered negatively, and 13% (79) gave an ambiguous answer and could not judge in either of the two hypotheses. The results are illustrated in Figure 8.

**Figure 9:**
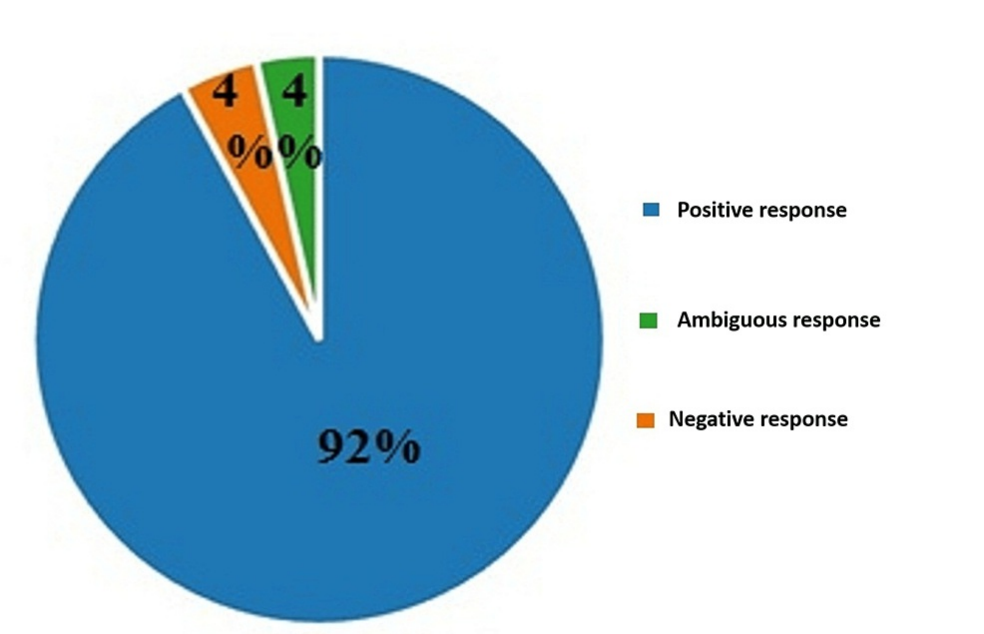
Answers by percentages to the ninth question: Do you think the right to life is the highest, inviolable, and absolute human right?

On the ninth question, "Do you think the right to life is the highest, inviolable, and absolute human right?" 92% (570) of the respondents answered positively. With an equal number of percentages, 4% responded negatively (27) and ambiguously (22). The results are illustrated in Figure 9.

**Figure 10:**
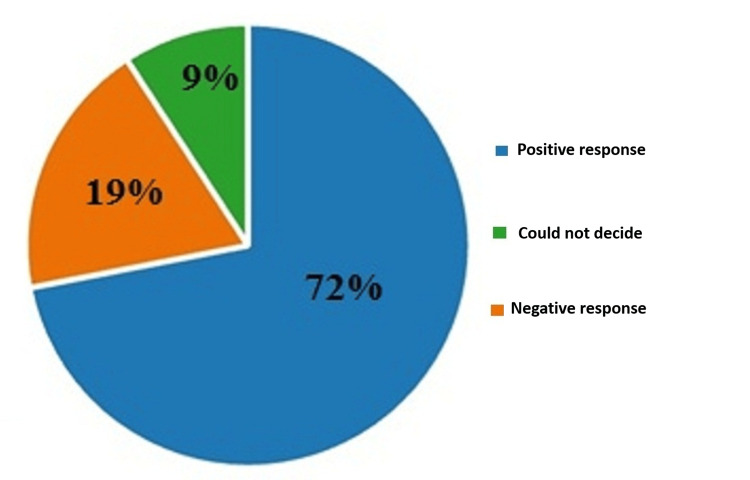
Answers by percentages to the tenth question: Do you think that a person should have the choice of when and in what way to end his life?

On the tenth question, whether a person should have the choice of when and how to end his life, 72% (446) answered positively, 19% (117) responded negatively, and 9% (57) could not decide. The results are illustrated in Figure 10.

**Figure 11:**
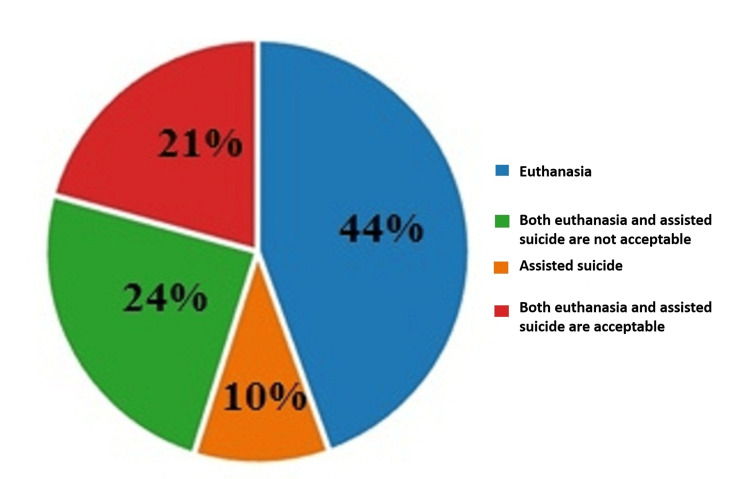
Answers by percentages to the eleventh question: What do you think is morally and legally permissible as a possible action?

On the last question of the questionnaire, about what is morally and legally permissible as a possible action, 44% (275) answered that it was euthanasia, 10% (64) responded that it was assisted suicide, 21% (128) of the respondents answered that both forms are acceptable, and 24% (151) of them shared their opinion that neither form is good. The results are illustrated in Figure 11.

## Discussion

The attitudes of humankind during the various stages of human history and regarding the eternal moral, ethical, and philosophical questions concerning life and death have a variable character. Over time, the polarization of opinions regarding the problem of euthanasia has become more and more clearly expressed in our modern society.

The topic arouses serious responses and interest in different countries and social strata [8-13]. The present research performed by surveying a targeted group of society (the medical community) in Bulgaria aims to bring clarity to the understanding of the problem of euthanasia, in-depth knowledge of the issues related to this problem, and the vision of the Bulgarian medical society regarding terminally ill patients, palliative medicine, and the level of development of medicine in Bulgaria in general.

The conclusions that can be drawn after this scientific analysis are that the medical community in Bulgaria has a clear view on the topic, which is still taboo for a significant part of our society.

The medical community in Bulgaria has a clear understanding of euthanasia as a matter and its different forms, and the majority of respondents believe that its legalization would not significantly change the relationship between the medical professional and his patient. For the most part, the respondents share the concern that the legalization of euthanasia would lead to the emergence of negative processes related to it, which is also observed in the countries where it is legally established [14-16].

At the same time, the medical community in Bulgaria is divided in its expert opinion regarding the quality of medical care, the treatment of terminally ill patients, and the possibility of such treatment in adequate volume and quality, so it is a natural alternative to euthanasia.

The same fact corresponds to the poor provision of the healthcare system in Bulgaria in terms of human, material, and technical resources as regards palliative medicine and care for terminally ill patients in the Republic of Bulgaria. Categorically, the medical community believes that the doctor should have an imperative priority in communication and the treatment-diagnostic process and that the patient, according to his views, is not mature enough and not prepared adequately to make his own decision about ending his life.

The same fact corresponds to the long-standing communication between doctor and patient in our health system, laid down as an unwritten rule in an autocratic style.

It is also interesting that despite the extremely negative attitude of various religions on the subject of euthanasia, a large part of the group, from the point of view of their religion, positively understands euthanasia and accepts it as a humane act of mercy.

However, the data analysis proves that our level of development of medicine has not yet reached that point in its development and progress to diagnose, adequately treat accurately, and give a clear assessment of the duration and quality of life for people in a terminal condition. It still has to develop so that it can be placed on the same plane as the discussion taking place around the legalization of euthanasia.

The majority of medical professionals clearly state their positive opinion on the adoption of a law to legalize euthanasia in the Republic of Bulgaria, clearly taking into account the fact that the right to life has always been and always will be the most absolute and fundamental human right. Contrary to the above, it is implied that it is inevitably linked to a quality and fulfilling life without suffering. Identically, they also answer that a person should have the right to know exactly when to end his own life.

The medical society in Bulgaria clearly shows its positive opinion regarding the idea that the different forms of euthanasia (active euthanasia and assisted suicide) should be defined as morally and legally permissible.

Our research confirms the Bulgarian medical community's opinion that the subject of the problems of euthanasia and its legal regulation is already ripe for public discussion, similar to many other European countries [2-7]. As two main antagonists in the legal-ethical peace, the right to a dignified life and the right to choose one's own life mark the dividing line of our society and set several urgent issues to be solved regarding the care of terminally ill patients and ensuring an adequate level of palliative care in Bulgaria.

The acceptance of euthanasia in several countries around the world creates a feeling among many activists that its acceptance in its entirety will lead to negative long-term consequences, leading to an increase in involuntary euthanasia and causing a severe upheaval in the most basic moral principles of humanity-principles related to humanity, human morality, and the basic principles of medical ethics.

Naturally, this should be a well-thought-out, gradual, comprehensive process of realizing the essence of the problem through a broad discussion involving the medical community as a whole and building working groups composed of narrowly profiled specialists from the fields of oncology, anesthesiology, intensive care, palliative medicine, emergency medicine, psychiatry, forensic medicine, etc. The essence of the topic should also go through a broad public discussion with citizens after a clear, purposeful, and open campaign to acquaint the citizens with the topic, with the depth of the problem, its advantages and disadvantages, and the experience in other countries, which is a "cornerstone" for its successful acceptance and implementation in society [17].

The actual limitations of the survey are the impossibility of assessing medical practitioners' opinions based on real cases of euthanasia in Bulgaria (this act in Bulgaria is still legally prohibited) and the impossibility of purely assessing the individual personal factor in each respondent as a person in routine human life.

## Conclusions

The actual problem of euthanasia as a conclusion raises several questions related to the process of acceptance of standard algorithms for action in such cases where the same action is legalized by law. It also includes the process of acceptance of strict regulations by the countries for the so-called negative phenomenon "death tourism" and several other administrative actions related to the mandatory registration of every case of euthanasia, the implementation of mandatory consultations with a psychiatrist and psychologist for patients seeking euthanasia as the only possible option, and providing possible alternatives regarding their illness. This is the unchangeable cornerstone for standardizing the legalization process and acceptance of "good death" in Bulgaria. In its essence, euthanasia creates both a social and an ethical conflict in our modern society, appearing at the same time as a kind of "stress test" for the health system.
